# Zein Nanoparticles-Loaded Flavonoids-Rich Fraction from *Fridericia platyphylla*: Potential Antileishmanial Applications

**DOI:** 10.3390/pharmaceutics16121603

**Published:** 2024-12-16

**Authors:** Monica Araujo das Neves, Caroline Martins de Jesus, Jhones Luiz de Oliveira, Samuel dos Santos Soares Buna, Lucilene Amorim Silva, Leonardo Fernandes Fraceto, Cláudia Quintino da Rocha

**Affiliations:** 1 PostGraduate Program in Chemistry, Center for Exact Sciences and Technology (CCET), UFMA-Federal University of Maranhão, São Luís 65080-805, Brazil; monica.neves@ufma.br (M.A.d.N.); samuel.buna@discente.ufma.br (S.d.S.S.B.); 2 PostGraduate Program in Health Sciences, Center for Biological and Health Sciences (CCBS), UFMA-Federal University of Maranhão, São Luís 65080-805, Brazil; caroline.mj@discente.ufma.br (C.M.d.J.); lucilene.silva@ufma.br (L.A.S.); 3 Department of Environmental Engineering, Institute of Science and Technology of Sorocaba, São Paulo State University (UNESP), Sorocaba 18087-180, Brazil; jhones.luiz@unesp.br (J.L.d.O.); leonardo.fraceto@unesp.br (L.F.F.)

**Keywords:** neglected diseases, brachydins, polymeric nanoparticle, zein, leishmaniasis

## Abstract

**Background/Objectives:** Leishmaniasis, caused by protozoa of the genus *Leishmania*, is a major global health issue due to the limitations of current treatments, which include low efficacy, high costs, and severe side effects. This study aimed to develop a more effective and less toxic therapy by utilizing zein nanoparticles (ZNPs) in combination with a nonpolar fraction (DCMF) from *Fridericia platyphylla* (Syn. *Arrabidaea brachypoda*), a plant rich in dimeric flavonoids called brachydins. **Methods:** Zein nanoparticles were used as carriers to encapsulate DCMF. The system was characterized by measuring particle diameter, polydispersity index, zeta potential, and encapsulation efficiency. Analytical techniques such as FTIR, DSC, and AFM were employed to confirm the encapsulation and stability of DCMF. Antileishmanial activity was assessed against *Leishmania amazonensis* promastigotes and amastigotes, while cytotoxicity was tested on RAW264.7 macrophages. **Results:** The ZNP-DCMF system exhibited favorable properties, including a particle diameter of 141 nm, a polydispersity index below 0.2, and a zeta potential of 11.3 mV. DCMF was encapsulated with an efficiency of 94.6% and remained stable for 49 days. In antileishmanial assays, ZNP-DCMF inhibited the viability of promastigotes with an IC50 of 36.33 μg/mL and amastigotes with an IC50 of 0.72 μg/mL, demonstrating higher selectivity (SI = 694.44) compared to DCMF alone (SI = 43.11). ZNP-DCMF was non-cytotoxic to RAW264.7 macrophages, with a CC50 > 500 μg/mL. **Conclusions:** Combining *F. platyphylla* DCMF with zein nanoparticles as a carrier presents a promising approach for leishmaniasis treatment, offering improved efficacy, reduced toxicity, and protection of bioactive compounds from degradation.

## 1. Introduction

Leishmaniasis is a neglected zoonotic disease caused by protozoan parasites of the genus *Leishmania*. This disease presents diverse clinical manifestations and epidemiological variations, impacting various geographic regions and contributing significantly to global morbidity and mortality. It is recognized as one of the six priority endemic diseases worldwide [1]. Leishmaniasis occurs in three primary forms: visceral, cutaneous, and mucocutaneous, with the latter affecting the mucous membranes of the upper respiratory tract, leading to severe tissue damage in the nose, mouth, and throat, often resulting in disfigurement [2].

According to the World Health Organization (WHO), approximately 700,000 to 1 million new cases of leishmaniasis occur annually, with 20,000 to 30,000 deaths attributed to its visceral and cutaneous forms, underscoring its relevance as a global public health issue [2]. In Brazil, most cases are concentrated in the North and Northeast regions, disproportionately affecting rural and low-income populations [3].

Current therapeutic strategies rely on pentavalent antimonials (e.g., meglumine antimoniate and sodium stibogluconate) and non-antimonial drugs (e.g., pentamidine, amphotericin B, paromomycin, and miltefosine). However, their clinical use could be improved by significant limitations, including drug resistance, high toxicity, and severe side effects [4,5,6,7].

The search for alternative treatments has highlighted the potential of medicinal plants as a source of bioactive compounds for drug development. The chemical diversity of plant-derived oils, extracts, and fractions provides valuable candidates for antiparasitic therapies. Numerous studies have demonstrated the efficacy of natural products against *Leishmania* species in vitro and in vivo [8,9]. Among secondary metabolites, alkaloids, steroids, terpenes, coumarins, and flavonoids exhibit promising activity and selectivity against these parasites [10,11,12].

In this context, *Fridericia platyphylla* (Syn. *Arrabidaea brachypoda*), commonly referred to as “cervejinha do campo” or “cipó-uma”, has emerged as a promising source of bioactive compounds. Phytochemical studies have identified a variety of secondary metabolites, including flavonoids, triterpenes, saponins, tannins, and polyphenols, in this species [13]. Traditionally, it has been used for the treatment of kidney stones and arthritis [14], while its antimicrobial [15], anti-inflammatory, antinociceptive properties [16], and activity against *Trypanosoma cruzi* [17] have also been reported. Additionally, its dichloromethane fraction (DCMF), containing dimeric flavonoids known as brachydins, has demonstrated antileishmanial [18] and antitumor activities [19,20,21].

Despite the potential of these plant-derived compounds, their application in therapeutics is constrained by challenges such as low water solubility, instability, and poor bioavailability, alongside potential side effects when they are used in their natural form [22]. Nanotechnology has emerged as a viable strategy to address these limitations by enhancing the physicochemical stability of bioactive compounds. Nanoencapsulation improves the bioavailability and efficacy of lipophilic substances while protecting them from degradation, thus providing novel solutions for leishmaniasis treatment [23,24,25].

Zein, a natural polymer derived from corn, has garnered attention due to its biocompatibility, biodegradability, and low toxicity. Its properties make it suitable for the development of micro/nanoparticles capable of encapsulating both hydrophilic and hydrophobic compounds [26,27].

This study presents an innovative approach that combines zein nanoparticles with the dichloromethane fraction (DCMF) of *Fridericia platyphylla*. This combination leverages zein nanoparticles’ structural and chemical properties to encapsulate nonpolar compounds such as brachydins, addressing the challenges of poor bioavailability and stability associated with plant-derived extracts. To our knowledge, no previous studies have explored this specific integration for leishmaniasis treatment. Encapsulation protects the active compounds from degradation and facilitates targeted delivery to infected cells, enhancing antiparasitic efficacy and reducing systemic toxicity.

Given these considerations, this study aimed to develop and characterize a zein-based nanostructured polymeric system incorporating the apolar DCMF of *Fridericia platyphylla*. The system was evaluated for its leishmanicidal activity against promastigote and amastigote forms of *L. amazonensis* and its cytotoxicity in RAW 264.7 macrophages in vitro.

## 2. Materials and Methods

### 2.1. Chemicals

Zein and Pluronic F-68 were obtained from Sigma-Aldrich (St. Louis, MO, USA), pentamidine (PTD) was purchased from Sigma-Aldrich (Munich, Germany) with a minimum purity of 98%. The cell culture reagents were obtained from Gibco/Invitrogen (Carlsbad, CA, USA). All other chemicals were obtained from Sigma-Aldrich (Munich, Germany), Merck (Munich, Germany) and Isofar (São Paulo, Brazil).

### 2.2. Plant Material

The roots of *F. platyphylla* were harvested in April 2021 at Sant’Ana da Serra farm, located in João Pinheiro, Minas Gerais, Brazil. Professor Maria Cristina Teixeira Braga Messias identified the plant species at the José Badine Herbarium, ICEB, Federal University of Ouro Preto. A voucher specimen (no. 17935) was deposited in the Herbarium of the Federal University of Ouro Preto, Brazil. The collection complied with Brazilian biodiversity protection regulations (SisGen No. A451DE4).

### 2.3. Extract Collection, Fractionation and Characterization

The extract and dichloromethane fraction (DCMF) were prepared following the procedure outlined by [17]. In summary, the roots of *F. platyphylla* were first dried and then ground using a knife mill. The resulting root powder underwent percolation with an ethanol/water mixture (7:3). After filtration, the ethanolic extract was collected and evaporated to dryness under reduced pressure at 40 °C to yield the crude extract. This extract was diluted in H_2_O: MeOH (7:3) and subjected to liquid–liquid partitioning to separate the dichloromethane (DCM) and water/methanol fractions. The dry DCM fraction was obtained by lyophilization after the removal of solvents from the DCM layer, resulting in a solid residue, then analyzed using high-performance liquid chromatography with photodiode array detection (HPLC-PDA) and liquid chromatography–mass spectrometry (LC-MS). The detailed chemical characterization methods for the fraction are provided in the work by [17].

### 2.4. Preparation of Zein Nanoparticles Loaded with DCMF

Zein nanoparticles (ZNP) were synthesized using the antisolvent precipitation method described by [28], with slight modifications. A hydroethanolic solution (85% *v*/*v*) containing 2% *w*/*v* of zein was prepared and stirred overnight. The solution was then purified by centrifugation for 30 min, followed by heating in a water bath for 15 min, and filtered through a 0.45 μm Millipore membrane to remove any insoluble particles. After filtration, 10 mg of DCMF was incorporated into 10 mL of the zein solution. In parallel, an aqueous solution of Pluronic F-68 (2% *w*/*v*) was prepared, and the pH was adjusted to 4. Using a 10 mL syringe, the zein–DCMF solution was rapidly injected into 30 mL of the Pluronic F-68 solution under magnetic stirring. The ethanol in the final colloidal dispersions was then removed using rotary evaporation at 40 °C (20 mL). The nanoparticle synthesis process was optimized to ensure improved particle size, polydispersity index, and encapsulation efficiency. The dichloromethane fraction (DCMF) concentration was optimized based on its solubility in the zein solution. Different DCMF concentrations (10 mg/mL, 5.0 mg/mL, 1.5 mg/mL, 1.0 mg/mL, and 0.5 mg/mL) were tested, with 0.5 mg/mL selected due to its superior stability. The resulting ZNP-DCMF nanoparticles were stored at 0.5 mg/mL concentration in an amber bottle at room temperature (25 °C).

### 2.5. Characterization of the Nanoparticle

The hydrodynamic diameter, polydispersity index, zeta potential and encapsulation efficiency of nanoformulation were monitored on days 0, 7, 14, 21, 28, 35, 42 and 49 of storage.

#### 2.5.1. Hydrodynamic Diameter, Polydispersity Index, and Zeta Potential

Photon correlation spectroscopy and microelectrophoresis were utilized to measure the nanoparticle systems’ hydrodynamic diameter, polydispersity index, and zeta potential. The samples were diluted in Milli-Q water and analyzed using a ZetaSizer ZS 90 (Malvern^®^) at a fixed angle of 90° and a temperature of 25 °C. The measurements were conducted in a volumetric cell of the NanoSight LM10 system, equipped with a 532 nm (green) wavelength laser, a CMOS camera, and NanoSight software (version 3.1). Each measurement was performed in triplicate.

#### 2.5.2. Encapsulation Efficiency Profile by HPLC-PDA

The amount of DCMF encapsulated in the nanoparticles was determined by the ultrafiltration/centrifugation method, in which the nanoparticle suspension was filtered using regenerated cellulose filters with a 10 kDa exclusion pore size (Microcon, Millipore), allowing only the passage of unencapsulated DCMF. HPLC quantified the DCMF in the ultrafiltrate. The amount of unencapsulated DCMF was obtained by subtracting the amount of DCMF detected in the ultrafiltrate (i.e., the fraction that was not encapsulated and, therefore, passed through the filter) from the total amount of DCMF initially added to the system (considered as 100%). In this way, it was possible to calculate the encapsulation efficiency of the nanoparticles, with encapsulated DCMF being considered as the fraction retained by the nanoparticles. Several studies have used this technique to determine the EE of active ingredients in nanocarriers [29,30]. Quantifying the three brachydins present in the DCMF, the profiles of ZN-DCMF and ZNP-WHITE were obtained using an Ultimate 3000 instrument (Thermo Fisher Scientific, Waltham, MA, USA). Chromeleon 7.2 software was used for the acquisition and interpretation of chromatograms. The mobile phase was H_2_O: MeOH acidified with 0.01% formic acid in gradient mode (ranging from 70% to 100% MeOH), with a Phenomenex Luna C18 column (150 × 4.60 mm, 5 μm). The injection volume was 100 μL and the detector wavelength was 240 nm. All tests were conducted in triplicate. Linear regression applied to the calibration curve points of the brachydins in the DCMF yielded the following equations: y = 31.593x − 1.8254 (r² = 0.9966 − BRA1); y = 88.744x − 12.261 (r² = 0.9944 − BRA2); and y = 211.63x + 28.32 (r² = 0.9903 − BRA3).

#### 2.5.3. Fourier Transform Infrared Spectroscopy (FTIR)

The nanoparticle formulations, both with and without DCMF, and the physical mixture of the nanoparticle components were analyzed using an attenuated total reflectance (ATR) accessory on a Varian^®^ FI-IR 660 spectrophotometer. The analysis was conducted across a wavenumber range of 400 to 4000 cm^−1^, with each sample undergoing 32 scans at a resolution of 8 cm^−1^.

#### 2.5.4. Differential Scanning Calorimetry (DSC)

Samples (~5 mg) of the nanoparticle formulations, both with and without DCMF, were placed in aluminum holders and analyzed using differential scanning calorimetry (DSC) with a Q20 model from TA Instruments. The analysis was conducted over a 25–300 °C temperature range, with a heating rate of 10 °C/min, under a nitrogen flow of 50 mL/min.

#### 2.5.5. Atomic Force Microscopy (AFM)

Atomic force microscopy was employed to verify the incorporation of nanoparticles into the polymeric gel matrix and examine the nanoparticles’ morphology. Samples containing nanoparticles embedded in the gel were prepared by spreading the undiluted formulation onto a silicon surface and allowing it to dry in a desiccator. The analysis was conducted using a BTO2217 microscope from Nanosurf, equipped with a ContAL-G-10 cantilever and a TapA1-G tip (BudgetSensors, Izgrev, Bulgaria), operating in intermittent contact mode at 90 Hz. The resulting images (256 × 256 pixels, TIFF format) were analyzed using the free Gwyddion software (http://gwyddion.net/) (accessed on 5 May 2024).

### 2.6. Biological Assay

#### 2.6.1. Maintenance of *L. amazonensis* Promastigotes

*L. amazonensis* (MHOM/BR/1987/BA-125) promastigotes were cultured in Schneider medium enriched with 10% fetal bovine serum (FBS), 50 μg/mL gentamicin, 0.4 g/L sodium bicarbonate, and 0.6 g/L calcium chloride, adjusted to a pH of 6.8. The culture was incubated at 27 °C in a Biochemical Oxygen Demand (BOD) incubator. For the experiments, promastigotes in the stationary growth phase (4 days of culture) exhibiting flagellar motility were used [31].

#### 2.6.2. Differentiation of Promastigote Forms of *L. amazonensis* in Axenic Amastigotes

To obtain axenic amastigotes, promastigotes (5.0 × 10^7^/mL) in the stationary phase were cultured in Schneider medium with a pH of 5.5, supplemented with 5% FBS, 50 μg/mL gentamicin, 0.4 g/L sodium bicarbonate, and 0.6 g/L calcium chloride. The culture was maintained at 32 °C in a BOD incubator for 96 h. Amastigote morphology was analyzed using Giemsa staining and microscopy (see Supplementary Material, Figure S1).

#### 2.6.3. In Vitro Leishmanicidal Activity

Promastigotes (5 × 10^7^ cells/mL) were treated with varying concentrations of DCMF (500 to 0.06 μg/mL) and ZNP-DCMF (125 to 0.06 μg/mL) in triplicate and incubated for 48 h at 26 °C in 100 μL of supplemented Schneider medium. The negative control included promastigotes cultured with only Schneider medium and 1% DMSO. At the same time, Pentamidine (50 to 1.56 μg/mL) served as the positive control, and ZNP without DCMF was used as the blank. For axenic amastigotes, parasites (1 × 10^6^/well) were treated with serial concentrations of DCMF (500 to 0.06 μg/mL) and ZNP-DCMF (125 to 0.06 μg/mL) in triplicate and incubated for 24 h at 32 °C in supplemented Schneider medium. The negative control was amastigotes in Schneider medium and 1% DMSO, Pentamidine (50 to 1.56 μg/mL) was the positive control, and ZNP without DCMF was the blank. Cytotoxicity was assessed by MTT assay.

#### 2.6.4. In Vitro RAW 264.7 Macrophage Cytotoxicity Assay

To assess cytotoxicity in normal cells, RAW 264.7 murine macrophages were cultured in a 96-well plate with the same concentrations of DCMF and ZNP-DCMF used for *L. amazonensis* promastigotes, in 100 μL of RPMI-1640 medium supplemented with 100 μg/mL penicillin, 100 U/mL streptomycin, 0.25 μg/mL amphotericin B, and 10% FBS. The assay was performed in triplicate with cells incubated at 37 °C and 5% CO_2_. The negative control comprised macrophages in a supplemented RPMI medium with 1% DMSO, while 100% DMSO was the positive control, and ZNP without DCMF served as the blank. Cell viability was measured using the MTT assay after 48 h.

#### 2.6.5. MTT Assay

The MTT assay determined cell viability for *L. amazonensis* promastigotes, axenic amastigotes, and RAW macrophages. Following incubation, the plates were centrifuged, and the supernatant was replaced with fresh medium containing MTT (5 mg/mL). The plates were incubated in a BOD incubator for 3 h, then centrifuged again, and the formazan crystals were dissolved in 100 μL of pure DMSO. Absorbance was measured at 540 nm using a microplate reader [32]. The selectivity index (S.I.) was calculated by dividing CC_50_ by IC_50_, where an S.I. greater than 1 indicates higher toxicity to the parasite, and an S.I. less than 1 indicates higher toxicity to the cell. A higher S.I. value reflects greater selectivity towards the parasite and reduced toxicity to the cell.

#### 2.6.6. Statistical Analysis

The software used for statistical analysis was GraphPad Prism 8.0 (GraphPad Inc., San Diego, CA, USA). Data were subjected to an analysis of variance (ANOVA) and differences between means were determined by Tukey’s test (*p* ≤ 0.05) to obtain the mean, IC_50_ and CC_50_.

## 3. Results and Discussion

### 3.1. Characterization and Stability of the Nanoparticle Containing DCMF

**Figure 1:**
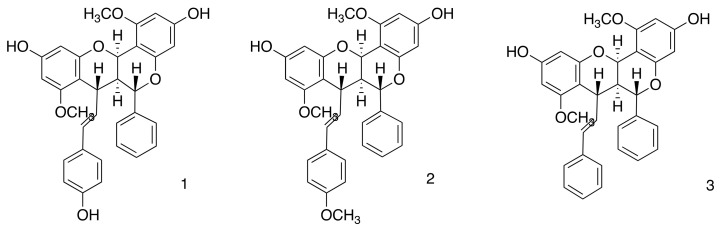
Chemical structure of brachydins present in the dichloromethane fraction (DCMF), (**1**) Brachydin 1 (BRA1), (**2**) Brachydin 2 (BRA2), and (**3**) Brachydin 3 (BRA3).

In this study, a zein nanoparticle was developed to create a carrier system for DCMF from *F. platyphylla*, containing the three dimeric flavonoids known to contain brachydins (Figure 1), thus offering a new alternative therapy to combat Leishmaniasis. The thorough chemical analysis of DCMF and the measurement of the brachydins within the fraction have been detailed in earlier research carried out by our team [17,33]. To verify the presence of brachydins in the DCMF, we conducted an HPLC-PDA analysis. We compared the results with the genuine brachydins standards isolated by our group (see Supplementary Material, Figure S2).

The antisolvent precipitation method was used to develop the system, introducing a hydroethanolic zein solution into an aqueous solution. Zein is an amphiphilic protein with hydrophobic and hydrophilic properties, facilitating its interaction with hydrophobic or partially hydrophobic compounds, such as bradykinins in the DCM fraction. It is soluble in binary solvents containing alcohol, and due to its high isoelectric point, there is a tendency for protein chains to aggregate in formulations with neutral or basic pH [28]. For this reason, using a surfactant is necessary to act as a stabilizing agent during manufacturing zein nanoparticles containing active ingredients like DCMF. Pluronic F-68 is an amphiphilic block copolymer composed of polyethylene glycol (PEG) and polypropylene glycol (PPG) chains, giving it hydrophilic and hydrophobic properties. Its main functions are to stabilize the nanoparticles and increase the solubility of bioactive compounds [34].

Table 1 shows the initial physical–chemical characterization of the zein nanoparticles with measurements of mean diameter (MD) measured by dynamic light scattering (DLS), polydispersity index (PDI), zeta potential (ZP), hydrogen potential (pH) and encapsulation efficiency (EE).

The results indicated that the addition of the fraction (ZNP-DCMF) led to an increase in the average diameter of the nanoparticles compared to the control nanoparticles (ZNPs), which can be attributed to the fact that, in general, the incorporation of active compounds into polymeric nanoparticles tends to promote an increase in particle diameter [35]. The control and DCMF nanoparticle showed polydispersity rates of less than 0.2. It can also be seen that the formulation containing DCMF showed zeta potential values of approximately 5.0 mV and both formulations showed similar pH values. In addition, the DCMF showed high interaction with the zein matrix, with encapsulation efficiency values of 99.8%.

**Figure 2:**
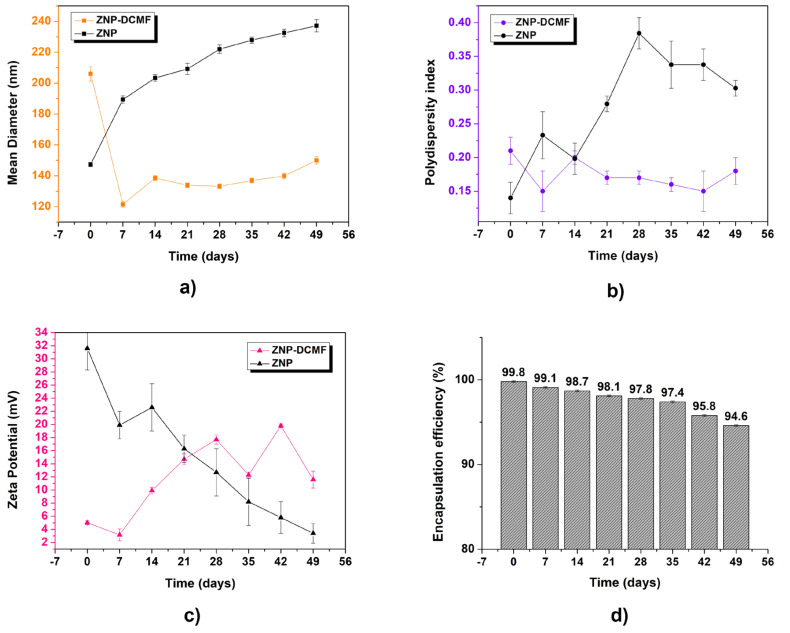
Characterization of control zein nanoparticles (ZNPs) and zein nanoparticles containing DCMF (ZNP-DCMF). (**a**) Mean diameter (nm) by DLS, (**b**) polydispersity index, (**c**) zeta potential (mV), and (**d**) encapsulation efficiency (%). The analyses were carried out for 49 days and all in triplicate at 25 °C.

To assess the stability of the formulations, the control nanoparticle (ZPN) and the nanoparticle containing DCMF (ZPN-DCMF) were analyzed according to storage time (Figure 2).

The ZPN and ZPN-DCMF average diameter results in Figure 2a show an increase as a function of storage time. However, it should be noted that the most significant increase in average particle diameter was observed for the control of nanoparticles compared to the nanoparticles containing DCMF. This could be attributed to the stabilizing effect of the presence of the active ingredient, which prevented the formation of aggregates. Similar results were observed by [36], who prepared zein nanoparticles to encapsulate eugenol and garlic essential oil. The formulation without the active ingredients showed a larger average diameter than the other nanoformulations, precipitating within 60 days of storage, while the latter could be analyzed over a more extended period. ZNP had an average diameter of 142 ± 1 nm at the start, and after 49 days, this value was 239 ± 3 nm. For ZNP-DCMF, the initial average diameter was 206 ± 4 nm, and after 49 days it was 150 ± 2 nm. No aggregate formation or precipitation of the system was observed throughout the storage period, and no significant changes were observed as a function of time, indicating that the formulation remained stable with an unimodal profile, i.e., the existence of only one type of nanocarrier population.

The polydispersity index results in Figure 2b show a significant increase in particle size for the ZNP over the period analyzed. This was not observed for the ZNP-DCMF, indicating a homogeneous and monodisperse particle size distribution. It should also be noted that these results corroborate those presented by the average diameter, since the control nanoparticles (ZNPs) showed a more significant change in values compared to those containing DCMF.

Regarding the zeta potential results (Figure 2c), ZNP showed a significant decrease in values (from 31.2 ± 1.1 mV to a final value of 21.8 ± 0.5 mV). At the same time, ZNP-DCMF exhibited a less pronounced increase in zeta potential over time, reaching approximately 11.6 ± 1.3 mV after 49 days, which is considered a moderate stability value for nanoscale formulations [37,38]. Although a moderately stable particle population was observed, falling outside the ideal stability range (±30 mV), the decrease in zeta potential in ZNP and ZNP-DCMF occurs for different reasons. In ZNP, the main reason is the absence of active ingredients, a trend also observed in the analysis of average diameter and PDI. In ZNP-DCMF, which contains the active ingredient rich in brachydins, the reduction is primarily due to a combination of hydrophobic interactions from nonpolar compounds present in this fraction, which reduce the surface charge density, and the effect of Pluronic, which masks these charges and stabilizes the particles through a steric barrier. This results in lower electrostatic repulsion between particles without necessarily compromising colloidal stability, which is maintained by the steric stabilization provided by Pluronic [39]. Therefore, the reduction in zeta potential does not necessarily indicate instability of the nanoparticle containing the DCMF.

The formulations’ encapsulation efficiency (Figure 2d) over time was analyzed to evaluate the stability of DCMF within the nanoparticles. Numerous factors influence the encapsulation rate of active ingredients in nanostructured systems, including the physicochemical properties of the active ingredient (e.g., solubility), the type of surfactant, polymer characteristics, pH of the medium, and the concentration of the active ingredient used [40,41]. Zein nanoparticles containing DCMF initially demonstrated an encapsulation efficiency of 99.8 ± 0.1%. Over 49 days of storage, a slight decrease of 5.2% was observed, with efficiency reaching 94.6 ± 0.1%. These results confirm the method’s effectiveness in maintaining stable nanoencapsulation of DCMF extracted from *Fridericia platyphylla* over time.

The high encapsulation efficiency of brachydins in zein nanoparticles is due to favorable physicochemical interactions. The hydrophobic regions of brachydins interact strongly with the hydrophobic core of zein, aiding their incorporation into the nanoparticle matrix. These interactions are further stabilized by hydrogen bonds with zein’s polar groups, van der Waals forces, and electrostatic interactions on the zein surface. Pluronic F-68, a non-ionic surfactant, enhances nanoparticle stability by forming a protective steric barrier, preventing aggregation and increasing homogeneity. Although Pluronic partially emulsifies some brachydins that are not fully encapsulated, this emulsification does not compromise the encapsulation efficiency. Encapsulated brachydins release slowly, while those interacting with Pluronic exhibit controlled release. This combination of interactions ensures high encapsulation efficiency (94.6%) and protects brachydins from degradation, enhancing the formulation’s stability and efficacy.

The results align with published studies using similar nanoparticle preparation methods. For instance, ref. [42] characterized zein nanoparticles stabilized with Pluronic to encapsulate geraniol and citronellal, achieving particle diameters of 80–200 nm, polydispersity indices > 0.3, zeta potentials between 10 and 33 mV, and encapsulation efficiencies > 90% over 120 days. Similarly, ref. [43] described zein nanoparticles containing eugenol and garlic essential oil, which exhibited high encapsulation efficiencies (>90%), spherical morphologies, average diameters of ~150 nm, polydispersity indices of 0.2, and zeta potentials of ~30 mV. These formulations protected active compounds from degradation, maintaining stability for over 90 days.

### 3.2. Characterization by Fourier Transform Infrared Spectroscopy (FTIR)

**Figure 3:**
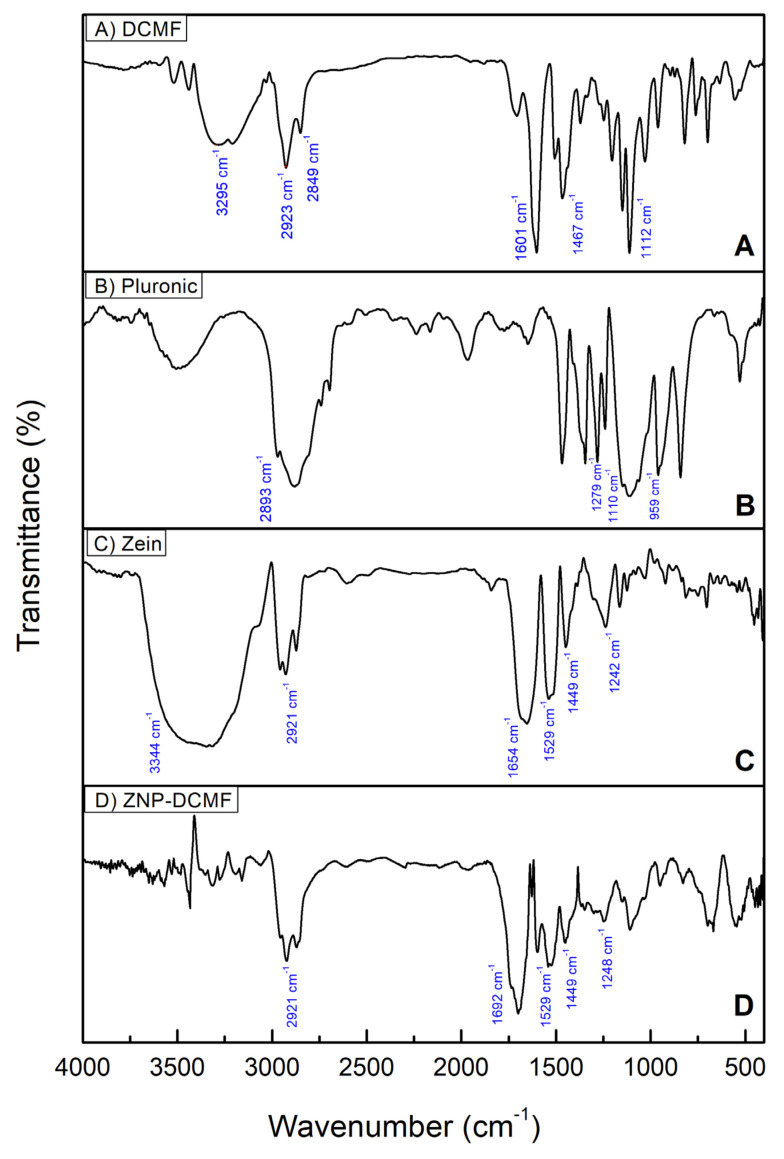
Characterization by FTIR using the ATR mode. (**A**) DCMF, (**B**) Pluronic F-68, (**C**) zein, and (**D**) ZNP-DCMF.

Infrared spectroscopy (FTIR) was used to investigate the interactions of DCMF with the zein nanoparticles and to determine whether the preparation process altered the components of the formulation. DCMF, the nanoparticle constituents (Zein and Pluronic F-68) and the nanoparticle loaded with DCMF were analyzed (Figure 3).

In Figure 3A, which refers to DCMF, specific bands alluding to the O-H groups of phenol in the 3295 cm^−1^ region stand out. Also noticeable in the 2849 and 2923 cm^−1^ regions are the C-H stretching vibrations of alkanes, while at 1601 and 1467 cm^−1^, the specific aromatic C=C vibrations stand out, as well as the CO bonds observed at 1112 cm^−1^. These data corroborate the NMR findings of [17] for the three brachydins in DCMF.

For the Pluronic (Figure 3B), the main bands were found at 2893 cm^−1^ corresponding to vibrations of the C-H bonds, at 959/1110 cm^−1^ stretching vibrations corresponding to the C-O bond (symmetrical and asymmetrical, respectively) of the ether group, and at 1279 cm^−1^ vibration corresponding to the CH_2_ group [44,45].

As expected, the spectrum of pure zein (Figure 3C) showed the characteristic bands corresponding to amides I, II, and III at 1654 (C=O stretch), 1529 (NH deformation and CN stretch), and 1242 cm^−1^, respectively. The peak at 3344 cm^−1^ corresponds to NH stretching. Also noticeable in the zein spectrum is the presence of a band referring to C-H bonds, which is in the spectral region at around 2921 cm^−1^, due to the presence of amino acids and fatty acids, as well as the presence of CH_2_ stretches, which are predominant in proteins and lipids, in the region between 1300 and 1500 cm^−1^ [46,47].

The FTIR results for ZNP-DCMF (Figure 3D) show that the bands characteristic of zein are present in the nanoparticle. In contrast, the bands characteristic of DCMF do not appear in the nanoparticle loaded with this active. This suggests that DCMF is encapsulated. In addition, the results indicate an interaction between the zein and the hydrophobic groups of the fraction, corroborating the findings of [32], who also used this technique to characterize zein nanoparticles loaded with geraniol and citronellal. In general, the FTIR results showed that no new bands appeared after the interaction between DCMF and the zein nanoparticle, indicating that the interactions were physical rather than chemical.

### 3.3. Characterization by Differential Scanning Calorimetry (DSC)

**Figure 4:**
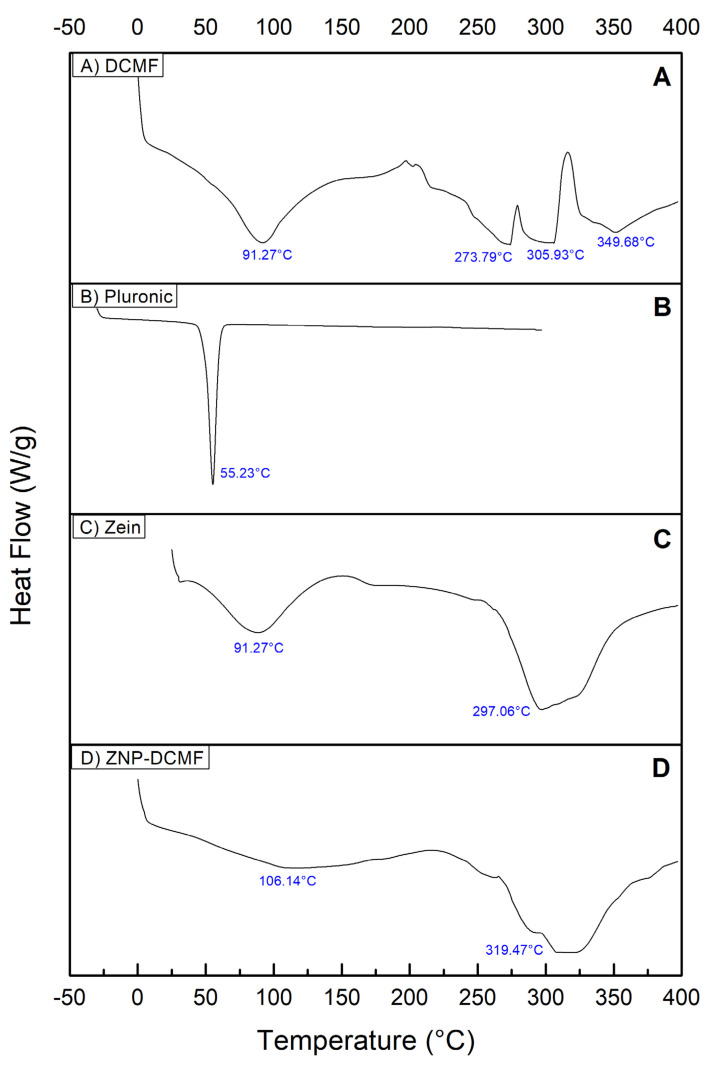
Characterization by differential scanning calorimetry (DSC). (**A**) DCMF, (**B**) Pluronic F-68, (**C**) zein and (**D**) ZNP-DCMF.

DSC analyses were carried out to better characterize and observe the interaction between DCMF and the zein nanoparticle. The results are shown in Figure 4, which shows the heat flow (W/g) as a function of temperature (°C).

The DSC curve for DCMF (Figure 4A) showed four endothermic thermal events. The first at 91.27 °C, is probably related to the loss of water and volatile constituents present in the sample [48]. The second, third and fourth thermal events occurred at 273.79 °C, 305.93 °C and 349.68 °C, probably related to the fusion of the three brachydins in DCMF. In the literature consulted, no references were found to DSC characterization studies of the plant species *F. platyphylla* and/or its fractions, making it impossible to compare their thermal behavior.

The thermogram of the Pluronic (Figure 4B) shows a well-defined, narrow and wide-ranging endothermic peak at 55.23 °C. Similar values were found by [49]. The thermogram of zein (Figure 4C) showed two broad, endothermic peaks, one at 91.27 °C, slightly higher than that found in the literature (73 °C, possibly due to protein hydration [27], and the second peak at 297.06 °C.

The DSC analysis of ZNP-DCMF (Figure 4D) showed peaks around 106.14 °C and 319.47 °C, which shows a shift in the endothermic peaks, evidencing a possible interaction between DCMF and the components of the nanoparticle [50], suggesting that the increase in the degradation temperature of zein/lectin particles loaded with curcumin is due to an increase in the hydrophobic effect and electrostatic interaction between the components of the particles. Similar results were found when the concentration of shellac was increased in the formation of zein nanoparticles. It was suggested that the nanoparticle size may better influence thermal stability [51]. The displacement of the endothermic peaks and the absence of peaks referring to DCMF also show no significant amounts of the fraction outside the nanoparticle, corroborating the results found in the encapsulation efficiency tests.

### 3.4. Atomic Force Microscopy (AFM)

**Figure 5:**
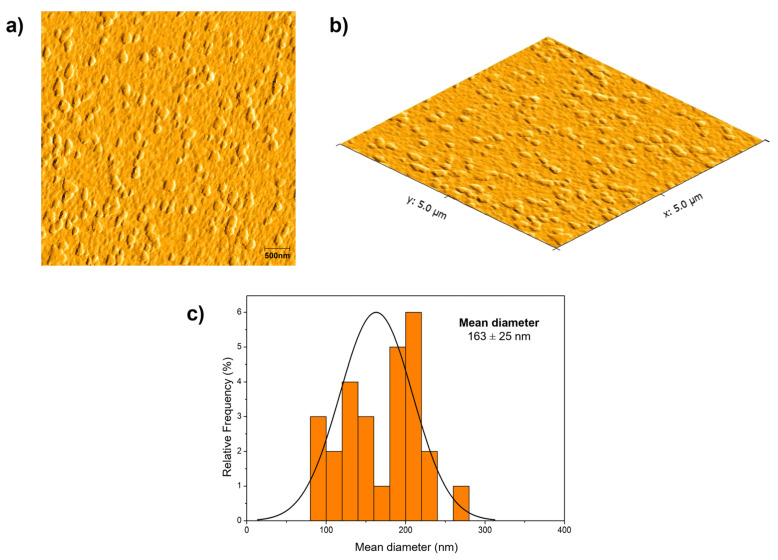
Micrographs obtained for nanoparticle using an atomic force microscope: (**a**) zein nanoparticles loading DCMF (2D image); (**b**) zein nanoparticles loading DCMF (3D image); (**c**) size distribution for zein nanoparticles loading DCMF. The analyses were performed using Gwyddion software.

Atomic force microscopy was used to check the morphological aspects of the zein nanoparticle containing DCMF. Figure 5 shows AFM micrographs of ZNP-DCMF. Analysis of the AFM micrographs of the zein nanoparticles with DCMF showed that the particles had a rounded appearance with a uniform size distribution, and no aggregates were observed. The average particle size was 163 ± 25 nm, like the DLS result in Figure 2. The average sizes found in this work are consistent with values reported for polymeric nanoparticles [41]. Ref. [43] reported similar sizes for zein nanoparticles loaded with eugenol and garlic essential oil, which ranged from 148 to 180 nm according to the amount of encapsulated oil and exhibited spherical shapes.

The results presented in Figure 2 and Figure 5 show a noticeable difference between the nanoparticle sizes obtained via DLS and AFM. DLS revealed an average hydrodynamic size of approximately 178 nm (mean over 49 days), with an average polydispersity index (PDI) of 0.15, indicating a relatively homogeneous size distribution in solution. Conversely, AFM images displayed more significant heterogeneity in the solid state, which can be attributed to the aggregation tendency of nanoparticles in this environment.

This aggregation can be explained by the intrinsic hydrophobicity of zein and the hydrophobic interactions of Pluronic F-68, which are key components of the formulation. Pluronic F-68 contributes to system stability through steric barriers that prevent aggregation, even with a relatively low mean zeta potential of 8.3 mV over the evaluated period. However, in the solid state, hydrophobic forces between nanoparticles become predominant, leading to aggregate formation and explaining the more significant morphological heterogeneity observed [52]

Thus, the differences between DLS and AFM reflect the distinct measurement environments: DLS evaluates nanoparticles dispersed in a liquid medium, while AFM captures the morphology of particles after drying. The observation of aggregates in the solid state does not contradict the DLS data but rather underscores the importance of considering interparticle interactions and environmental conditions when designing controlled release systems.

### 3.5. Nanoparticles Containing DCMF from F. platyphylla Show Antileishmanial Activity In Vitro

Table 2 presents the results of the activity of the pentamidine (PNT), the fraction (DCMF), and zein nanoparticles loaded with the fraction (ZNP-DCMF) against *L. amazonensis*. Antiparasitic activity against promastigote forms was concentration-dependent for DCMF (IC_50_ = 253.1 μg/mL) and ZNP-DCMF (IC_50_ = 36.33 μg/mL) (see Supplementary Material, Figure S3). Interestingly, the IC_50_ of ZNP-DCMF was significantly lower than that of DCMF against *L. amazonensis*. Similar results were found when DCMF and ZNP-DCMF were tested against axenic amastigote forms (IC_50_ of 6.96 μg/mL and 0.72 μg/mL, respectively), indicating reduced parasite viability (see Supplementary Material, Figure S4). DCMF demonstrated a reduction comparable to that of the standard drug (PNT); however, ZNP-DCMF exhibited the lowest IC_50_ value for amastigote forms.

**Figure 6:**
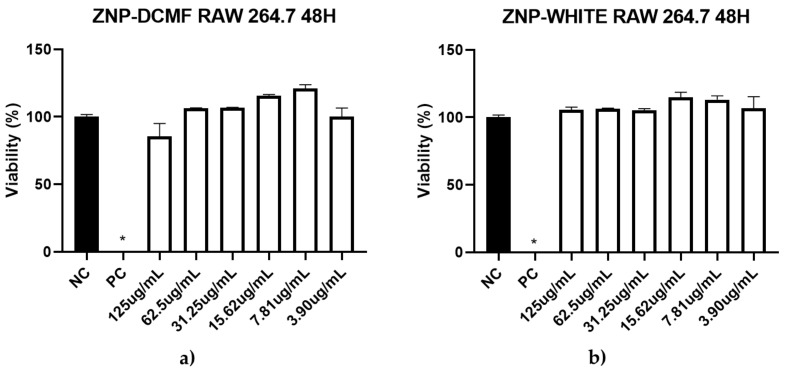
Cytotoxic activity of ZNP-DCMF (**a**) and ZNP-WHITE (**b**) against RAW 264.7 macrophages strain treated for 48 h. NC = negative control; PC = positive control; * asterisks indicate statistically significant differences to the negative control at *p* < 0.05.

Additionally, in this study, a cytotoxicity assay on RAW 264.7 macrophages showed that ZNP-DCMF exhibited no toxicity (CC_50_ > 500 μg/mL), demonstrating high selectivity for the parasite in both forms (Figure 6). Pentamidine was the most toxic compound (CC_50_ = 178.0 μg/mL). The selectivity index (S.I. = CC_50_/IC_50_) is a value that relates activity to cytotoxicity. The higher the selectivity index (>1), the more selective the drug is for the parasite [53]. Surprisingly, the selectivity index value increased when DCMF was encapsulated in zein nanoparticles, rising from 1.18 to 13.76 for promastigote forms and 43.11 to 694.4 for axenic amastigotes, respectively. Its action against the obligatory intracellular form (amastigote) in the vertebrate host underscores its clinical significance and potential benefits, as it can reduce parasitic load within cells, inhibiting infection progression.

Over the past decade, drug delivery systems based on natural or synthetic polymers have been widely used to enhance the efficacy of leishmaniasis treatment [8]. Similarly, combining nanoparticles with natural products with leishmanicidal potential has been researched to develop new therapies. These nanocarriers offer the advantage of penetrating cells such as macrophages and reaching the infectious parasite, enabling targeted and efficient drug release [54,55]. Moreover, natural compounds have shown the ability to induce changes in cellular structures, including at the mitochondrial level, resulting in the death of promastigote and intracellular amastigote forms [56,57]. However, it is noteworthy that there have yet to be any previous studies on developing zein nanoparticles for releasing active ingredients present in DCMF from the plant species *Fridericia platyphylla*. Thus, this study presents promising new perspectives for the application of nanoparticles in combating leishmaniasis.

## 4. Conclusions

In conclusion, developing a zein nanoparticle system encapsulating the active nonpolar fraction (DCMF) of *Fridericia platyphylla* demonstrated significant antileishmanial activity with reduced cytotoxicity and enhanced selectivity. This innovative approach improves the therapeutic efficacy and protects the bioactive compounds from degradation, offering a promising and sustainable alternative for treating leishmaniasis with fewer side effects. Integrating natural compounds with nanotechnology presents a valuable strategy for advancing leishmaniasis treatment, although further studies are needed to assess its clinical safety and efficacy.

## Figures and Tables

**Table 1:** Physicochemical characterization of the control nanoparticle (ZNP) and nanoparticle loaded with DCMF (ZNP-DCMF).

Samples	DLS (nm)	PDI	ZP (mV)	pH	EE%
ZNP	142 ± 1	0.185 ± 0.02	31.2 ± 1.1	4.01 ± 0.01	-
ZNP-DCMF	206 ± 4	0.207 ± 0.05	5.0 ± 0.3	4.07 ± 0.02	99.8 ± 0.1

The parameters analyzed were the mean diameter (MD) measured by dynamic light scattering (DLS), polydispersity index (PDI), zeta potential (ZP), potential hydrogenionic (pH) and encapsulation efficiency (EE). ZNP: Empty nanoparticles. ZNP-DCMF: Nanoparticles loaded with DCMF. -: Analysis not increased as mean ± standard deviation (*n* = 3).

**Table 2:** Inhibitory concentration detected in promastigote and axenic amastigote forms of *L. amazonensis* and cytotoxic concentration in RAW 264.7 macrophages treated with PNT, DCMF and ZNP-DCMF.

Promastigote			
Samples	IC_50_ (μg/mL)	CC_50_ (μg/mL)	S.I.
PNT	4.0	178.0	44.50
DCMF	253.1	300.1	1.18
ZNP-DCMF	36.33	>500	13.76
**Amastigote**			
PNT	5.0	178.0	35.60
DCMF	6.96	300.1	43.11
ZNP-DCMF	0.72	>500	694.44

PNT: pentamidine; DCMF: dichloromethane fraction; ZNP-DCMF: zein nanoparticles loading DCMF; CC_50_: cytotoxic concentration to 50% of macrophages; IC_50_: cytotoxic concentration to 50% of parasites; S.I.: selectivity index (CC_50_/IC_50_).

## Data Availability

Data are contained within the article and Supplementary Materials.

## Supplementary Materials

The following supporting information can be downloaded at https://www.mdpi.com/article/10.3390/pharmaceutics16121603/s1, Figure S1. They are obtaining axenic amastigote forms. The culture of axenic amastigotes was obtained from an early stationary phase culture of *Leishmania amazonensis* promastigotes observed under light microscopy. (a) Promastigotes form in the stationary phase; (b) forms amastigote on day 4 of culture without externalized flagellum. Reference bar: 25 μm. Figure S2. a) Chromatographic profile of DCMF (A), ZNP-DCMF (B) and ZNP-WHITE, obtained by HPLC-PDA, and chemical structure of the compounds Brachydin A (1), Brachydin B (2) and Brachydin C. Figure S3. Characterization by FTIR using the ATR mode. (A) DCMF, (B) Pluronic F-68, (C) Zein and (D) ZNP-DCMF. Figure S4. Characterization by differential scanning calorimetry (DSC). (A) DCMF, (B) Pluronic F-68, (C) Zein and (D) ZNP-DCMF. Figure S5. Viability of *L.* (*L*) *amazonensis* promastigotes forms treated for 48 h with DCMF (a) ZNP-DCMF (b) and ZNP-WHITE (c). NC = negative control; PC = positive control; * asterisks indicate statistically significant differences about the negative control at *p* < 0.05. Figure S6. Viability of axenic *L.* (*L*) *amazonensis* amastigote forms treated for 48 h with DCMF (a) ZNP-DCMF (b) and ZNP-WHITE (c). NC = negative control; PC = positive control; * asterisks indicate statistically significant differences in relation to the negative control at *p* < 0.05.
