# Supplementary material for: Zein Nanoparticles-Loaded Flavonoids-Rich Fraction from Fridericia platyphylla: Potential Antileishmanial Applications
Source: Pharmaceutics. 2024 Dec 16;16(12):1603. doi: 10.3390/pharmaceutics16121603 (PMC11678320; doi:10.3390/pharmaceutics16121603)
Supplement: Supplementary file 1 [file pharmaceutics-16-01603-s001.zip › pharmaceutics-3331594-supplementary.pdf]

SUPPLEMENTARY MATERIAL

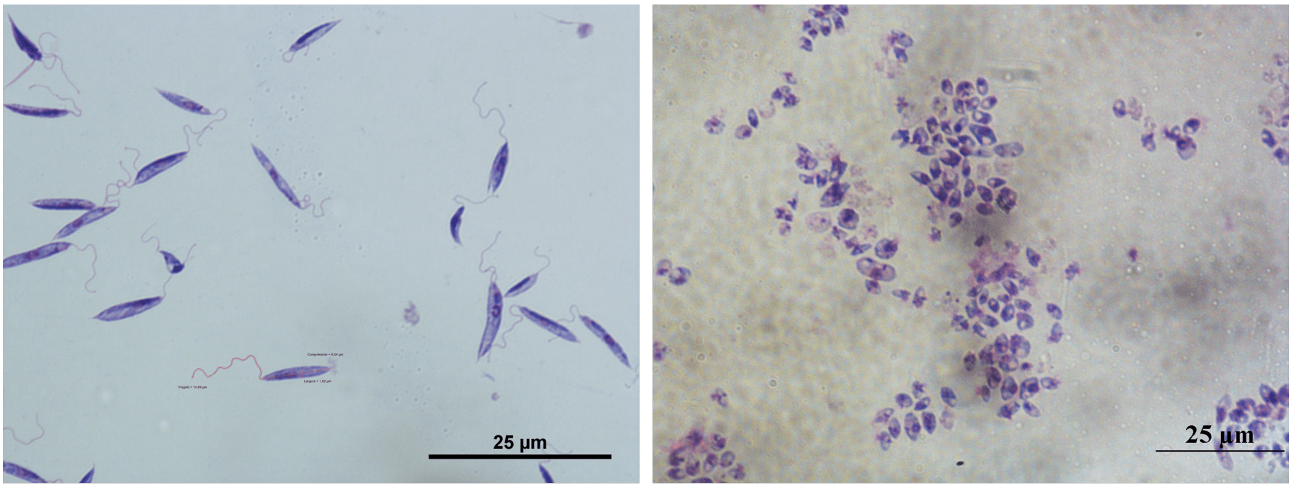

a) b)

Figure S1. Obtaining axenic amastigote forms. The culture of axenic amastigotes was obtained from an early stationary phase culture of *Leishmania amazonensis* promastigotes observed under light microscopy. (a) Promastigotes form in stationary phase; (b) Forms amastigote on day 4 of culture without externalized flagellum—reference bar: 25 µm.

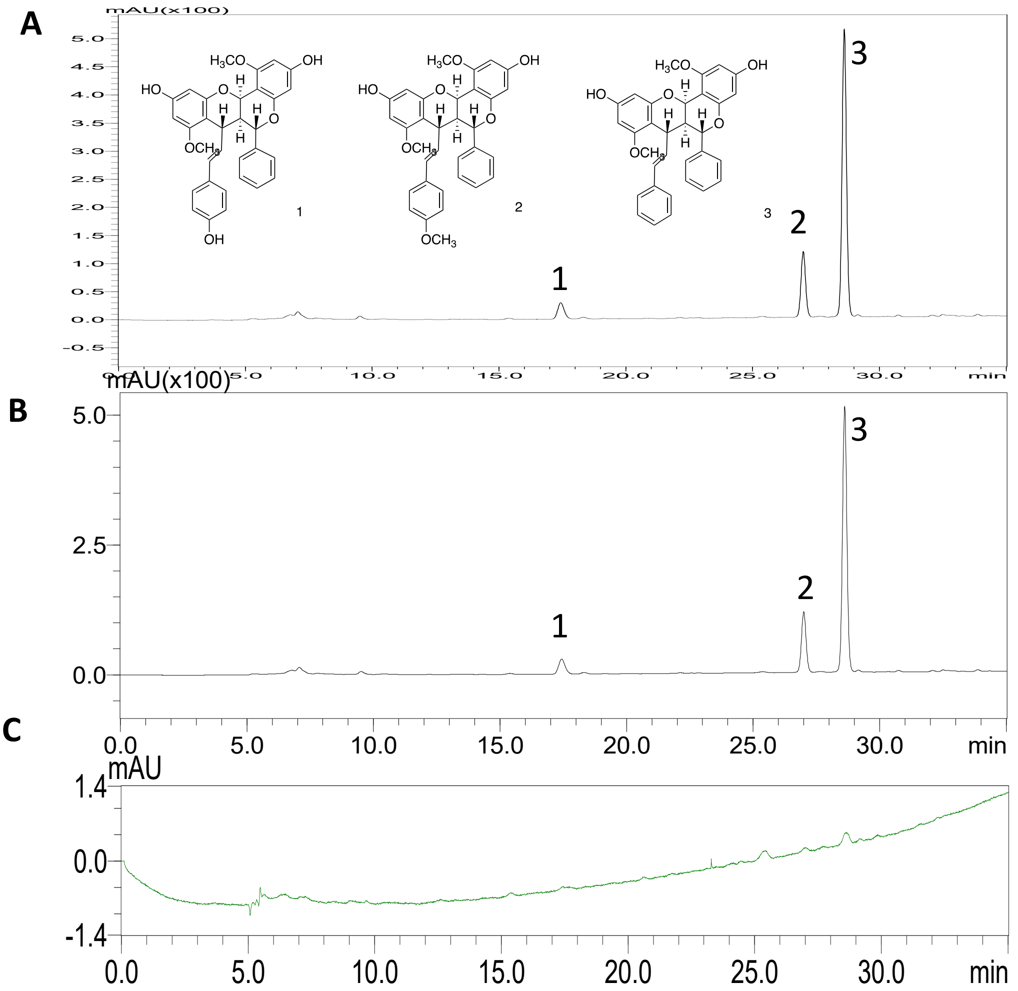

Figure S2. Chromatographic profile of DCMF (A), ZNP-DCMF (B) and ZNP-WHITE, obtained by HPLC-PDA, and chemical structure of the compounds brachydin A (1), brachydin B (2) and brachydin C (3).

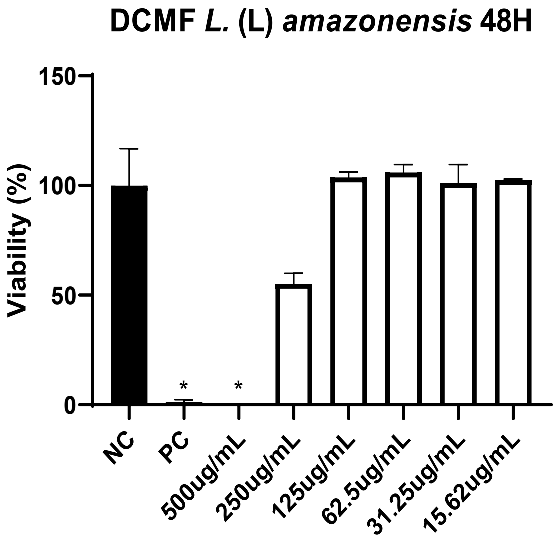

a)

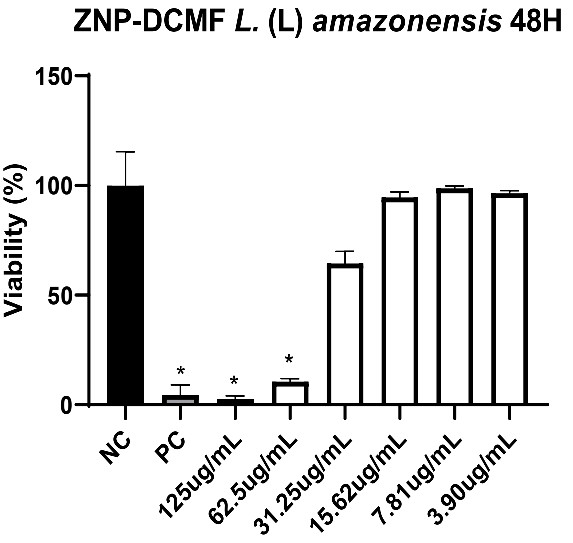

b)

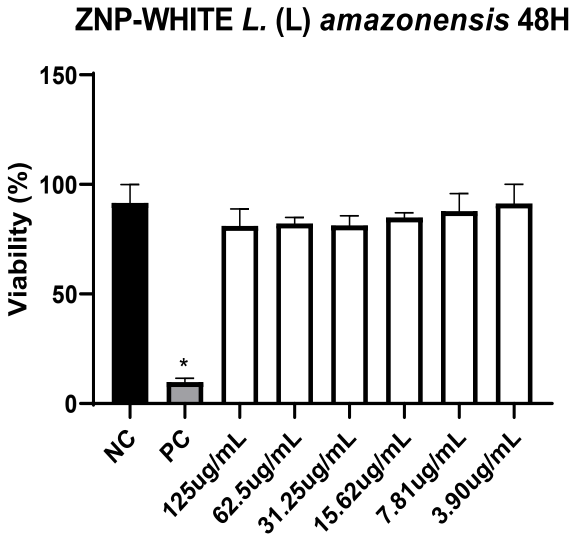

c)

Figure S3. Viability of *L. (L) amazonensis* promastigotes forms treated for 48 h with DCMF (a) ZNP-DCMF (b) and ZNP-WHITE (c). NC = negative control; PC= positive control; *Asterisks indicate statistically significant differences with the negative control at $p < 0.05$.

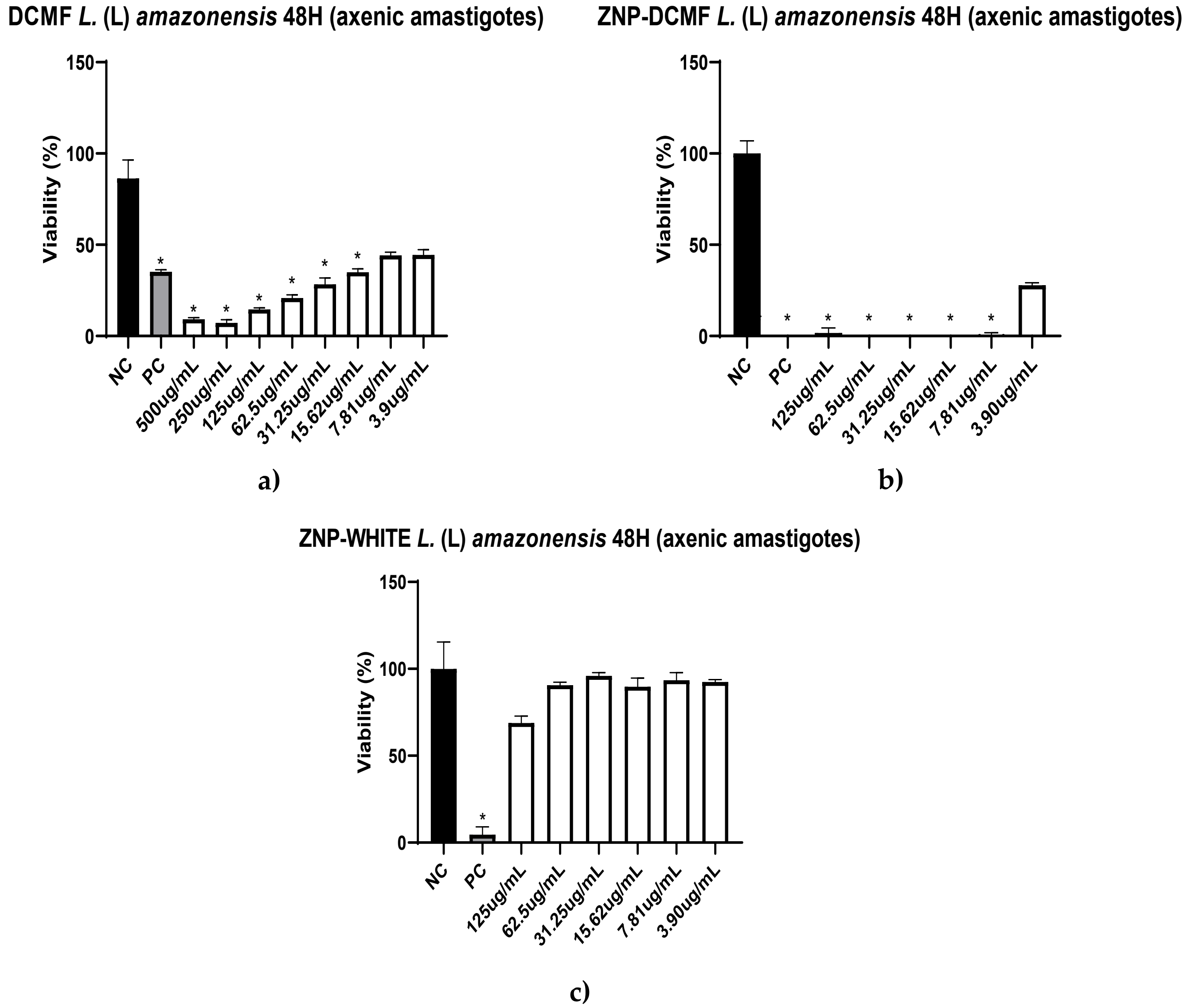

Figure S4. Viability of axenic *L. (L) amazonensis* amastigote forms treated for 48 h with DCMF (a) ZNP-DCMF (b) and ZNP-WHITE (c). NC = negative control; PC= positive control; *Asterisks indicate statistically significant differences to the negative control at $p < 0.05$
